# MicroRNA-137-3p Improves Nonalcoholic Fatty Liver Disease through Activating AMPK*α*

**DOI:** 10.1155/2021/4853355

**Published:** 2021-12-29

**Authors:** Yuanjie Yu, Chunping He, Shiyun Tan, Mengjun Huang, Yitian Guo, Ming Li, Qian Zhang

**Affiliations:** ^1^Department of Gastroenterology, Renmin Hospital of Wuhan University, Wuhan 430060, Hubei, China; ^2^Hubei Key Laboratory of Digestive System Disease, Renmin Hospital of Wuhan University, Wuhan 430060, Hubei, China; ^3^Department of Nutrition, The Central Hospital of Wuhan, Tongji Medical College, Huazhong University of Science and Technology, Wuhan 430014, Hubei, China; ^4^Department of Infectious Diseases, Renmin Hospital of Wuhan University, Wuhan 430060, Hubei, China

## Abstract

Nonalcoholic fatty liver disease (NAFLD) is one of the most common chronic liver diseases worldwide and can develop to nonalcoholic steatohepatitis and later hepatic cirrhosis with a high prevalence to hepatocellular carcinoma. Oxidative stress and chronic hepatic inflammation are implicated in the pathogenesis of NAFLD. MicroRNA-137-3p (miR-137-3p) are associated with oxidative stress and inflammation; however, its role and mechanism in NAFLD remain unclear. Mice were fed with a high-fat diet (HFD) for 24 weeks to establish the NAFLD model. To overexpress or suppress hepatic miR-137-3p expression, mice were intraperitoneally injected with the agomir, antagomir, or respective controls of miR-137-3p at a dose of 100 mg/kg weekly for 6 consecutive weeks before the mice were sacrificed. To validate the involvement of AMP-activated protein kinase alpha (AMPK*α*) or cAMP-specific phosphodiesterase 4D (PDE4D), HFD mice were intraperitoneally injected with 20 mg/kg compound C or 0.5 mg/kg rolipram every other day for 8 consecutive weeks before the mice were sacrificed. Hepatic miR-137-3p expression was significantly decreased in mice upon HFD stimulation. miR-137-3p agomir alleviated, while miR-137-3p antagomir facilitated HFD-induced oxidative stress, inflammation, and hepatic dysfunction in mice. Mechanistically, we revealed that miR-137-3p is directly bound to the 3′-untranslated region of PDE4D and subsequently increased hepatic cAMP level and protein kinase A activity, thereby activating the downstream AMPK*α* pathway. In summary, miR-137-3p improves NAFLD through activating AMPK*α* and it is a promising therapeutic candidate to treat NAFLD.

## 1. Introduction

Nonalcoholic fatty liver disease (NAFLD) is characterized as metabolic disorder and hepatic steatosis and emerges as one of the most common chronic liver diseases worldwide that can develop to nonalcoholic steatohepatitis (NASH) and later hepatic cirrhosis with a high prevalence to hepatocellular carcinoma [1–4]. Multiple factors are implicated in the pathogenesis of NAFLD, including oxidative stress and chronic hepatic inflammation [5, 6]. During the simple hepatic steatosis stage, overwhelming nonestesterified fatty acids (NEFAs) in the hepatocytes destroy the electron transport chain and impair mitochondrial function, eventually leading to the accumulation of excessive reactive oxygen species (ROS) [7]. In addition, these NEFAs and the intracellular substances released from the injured hepatocytes also activate the intrahepatic Kupffer cells, recruit the infiltration of other leukocytes, and generate a proinflammatory microenvironment, thereby facilitating the progression of NAFLD. Therefore, it is reasonable to treat NAFLD via suppressing oxidative stress and inflammation.

AMP-activated protein kinase alpha (AMPK*α*) is a highly conserved energy sensor in eukaryotic cells, and AMPK*α* activation has become an attractive strategy to treat metabolic diseases [8, 9]. Beyond the function in maintaining intracellular energy homeostasis, AMPK*α* also plays critical roles in modulating oxidative stress and inflammation [10]. Hu et al. showed that AMPK*α* activation upregulated the expression of uncoupling protein 2 and thus restrained myocardial ROS generation [11]. Nuclear factor erythroid-2-related factor-2 (NRF2) is identified as a core regulator of antioxidant defenses through binding to the antioxidant responsive elements of various antioxidant genes, including glutathione (GSH), superoxide dismutase (SOD), and catalase (CAT) [12]. Qu et al. showed that AMPK*α* activation by gastrodin increased the expression and nuclear translocation of intrahepatic NRF2 and subsequently ameliorated oxidative stress and inflammatory response in NAFLD rodents [13]. A complex molecular network is orchestrated to converge on nuclear factor-kappa B (NF-*κ*B) to trigger hepatic inflammation upon different stresses [14]. Zhang et al. reported that NF-*κ*B was robustly activated in hepatocytes and the liver upon NAFLD stimuli, which in turn aggravated hepatic damage via promoting the generation of multiple proinflammatory cytokines. Intriguingly, AMPK*α* activation could block NF-*κ*B transcription activity and attenuate hepatic inflammation and fibrosis [15]. Particularly, genetic liver-specific AMPK*α* activation significantly reduced hepatic steatosis and inflammation, thereby alleviating high-fat diet- (HFD-) induced NAFLD [16]. These findings identify AMPK*α* as a promising therapeutic candidate for NAFLD.

MicroRNAs (miRNAs) are kinds of endogenously expressed, single-stranded, small noncoding RNAs and involve in multiple biological processes through binding to the 3′-untranslated region (UTR) of messenger RNAs for degradation or translation repression [17–19]. Various miRNAs are associated with intracellular ROS generation and inflammatory response and have been implicated in the development of NAFLD [20, 21]. miR-378 expression was elevated in the liver of dietary obese mice and NASH patients, and this elevation facilitated hepatic inflammation and fibrosis in mice [15]. Hanin et al. reported that hepatic miR-132 level was dramatically increased in NAFLD patients and mice and that the transgenic mice with miR-132 overexpression exhibited a more severe fatty liver phenotype and metabolic syndrome at basal conditions [22]. miR-137-3p is widely expressed across species and organs and also enriched in the liver. Tian et al. recently determined that miR-137-3p is directly bound to the 3′-UTR of Src to inactivate the downstream mitogen-activated protein kinase (MAPK) pathway, thereby preventing inflammatory response, oxidative stress, and neuronal injury in ischemic stroke [23]. Studies on the role of miR-137-3p in hepatic diseases are primarily confined to hepatocellular carcinoma [24, 25]. A previous study based on miRNA transcriptome sequencing identified miR-137-3p as a promising antioxidant target in the liver [26]. In the current study, we tried to investigate the role and molecular basis of miR-137-3p in NAFLD.

## 2. Materials and Methods

### 2.1. Animals and Study Design

Two hundred eight-week-age male C57BL/6 mice were purchased from Huafukang Bioscience Co., Ltd. (Beijing, China) and adaptively fed in a SPF barrier facility with free access to water and normal diet (ND, 10% kcal fat, 70% kcal carbohydrates, and 20% kcal protein) for 1 week before the study commenced. The NAFLD model was established by feeding mice with a HFD (60% kcal fat, 20% kcal carbohydrates, and 20% kcal protein) for 24 weeks, whereas the matched mice were maintained on a ND [27]. To overexpress or suppress hepatic miR-137-3p expression, mice were intraperitoneally injected with the agomir, antagomir, or respective controls of miR-137-3p at a dose of 100 mg/kg weekly for 6 consecutive weeks before the mice were sacrificed. The miR-137-3p agomir (#miR40000149-4-5), antagomir (#miR30000149-4-5), and respective controls were purchased from Guangzhou RiboBio Co., Ltd. (Guangzhou, China). To validate the involvement of AMPK*α* or cAMP-specific phosphodiesterase 4D (PDE4D), HFD mice were intraperitoneally injected with 20 mg/kg compound C (CpC, #171260; Sigma, St. Louis, MO, USA), 0.5 mg/kg rolipram (#R6520; Sigma), or an equal volume of vehicle every other day for 8 consecutive weeks before the mice were sacrificed [28, 29]. All mice were euthanatized by cervical dislocation. The animal experiments were approved by the Animal Care and Use Committee of Renmin Hospital of Wuhan University and were also in compliance with the *Animal Research: Reporting of In Vivo Experiments* guidelines.

### 2.2. Biochemical Analysis

Fasting blood glucose (FBG) and serum insulin levels were measured using a OneTouch UltraEasy glucometer (LifeScan, Wayne, PA, USA) and the commercial ELISA kit (#EMINS; ThermoFisher Scientific, Waltham, MA, USA), respectively, and the homeostatic model assessment-insulin resistance (HOMA-IR) index was calculated using the formula: FBG × fasting serum insulin/22.5. Serum triglyceride (TG), total cholesterol (TC), alanine transaminase (ALT), and aspartate transaminase (AST) were determined by a Beckman automatic biochemistry analyzer (Palo Alto, CA, USA) according to the manufacturer's instructions. Hepatic TG (#290-63701), TC (294-65801), and NEFA (#294-63601) levels were measured using the commercial kits from Wako (Osaka, Japan). Hydroxyproline is a major component of collagen, and hepatic hydroxyproline levels were thus determined to evaluate fibrotic remodeling using a commercial kit (#MAK008, Sigma). Briefly, fresh livers were homogenized and hydrolysed in hydrochloric acid (HCl, 12 mol/L) at 120°C for 3 h, which were then incubated with chloramine T and 4-(dimethylamino) benzaldehyde, with the absorbance measured at 560 nm. Hepatic ROS generation was determined by a 2′,7′-dichlorodi-hydrofluorescein diacetate (DCFH-DA) probe as previously described [30, 31]. In brief, fresh livers were homogenized and incubated with 50 *μ*mol/L DCFH-DA (#D6883, Sigma) at 37°C for 1 h in the dark, and then, the fluorescent intensities were determined at the excitation/emission wavelength of 485/535 nm. Hepatic hydrogen peroxide (H_2_O_2_, #S0038; Beyotime, Shanghai, China) level, malondialdehyde (MDA, #S0131S; Beyotime) level, GSH (#S0052; Beyotime) level, total SOD activity (#S0101S; Beyotime), and CAT activity (#S0051; Beyotime) were all determined by the commercial kits according to the manufacturer's instructions. The inflammatory cytokines, including interleukin-1 beta (IL-1*β*, #ab197742; Abcam, Cambridge, UK), IL-6 (#ab222503; Abcam), monocyte chemotactic protein-1 (MCP-1, #ab208979; Abcam), tumor necrosis factor-alpha (TNF-*α*, #ab208348; Abcam), and IL-10 (#ab108870; Abcam), were measured by the ELISA method. Hepatic cAMP levels were determined by a competitive cAMP ELISA kit (#ab65355; Abcam) according to the manufacturer's instructions. Briefly, fresh livers were homogenized and incubated with the Acetylating Reagent Mix at room temperature for 10 min to acetylate cAMP, which was then added to the protein G coated 96-well plate, followed by the reconstituted cAMP antibody. Next, horseradish peroxidase- (HRP-) conjugated cAMP was added and the optical density was detected at 450 nm. Hepatic protein kinase A (PKA) activity was detected using the PKA Kinase Activity Assay kit (#ab139435; Abcam) based on a synthetic PKA-specific substrate. For the determination of adenyl cyclase (AC) activity, fresh livers were homogenized and incubated with adenosine triphosphate at 37°C for 30 min, and then, the hepatic cAMP levels were determined and normalized to the total protein concentration to calculate the relative AC activity [32].

### 2.3. Western Blot

Liver tissues and primary hepatocytes were harvested and lysed in RIPA buffer (#G2002; Servicebio, Wuhan, China) with the protein concentrations measured by the Pierce™ BCA Protein Assay kit (#23225; ThermoFisher Scientific) [33–35]. Next, equal amounts of total proteins were separated by SDS-PAGE and transferred onto PVDF membranes that were then blocked with 5% skimmed milk at room temperature for 1 h and probed with indicating primary antibodies at 4°C overnight. The primary antibodies against NRF2 (#ab92946), *β*-actin (#ab8226), phospho-NF-*κ*B p65 (p-p65, #ab76302), and total-p65 (t-p65, #ab32536) were purchased from Abcam, while anti-p-AMPK*α* (#50081) and anti-t-AMPK*α* (#5832) were purchased from Cell Signalling Technology (Beverly, MA, USA). Anti-PDE4D (#12918-1-AP) was purchased from Proteintech (Rosemont, IL, USA). On the second day, the membranes were incubated with the HRP-conjugated secondary antibodies at room temperature for 1 h. Subsequently, the protein bands were visualized by LumiGLO chemiluminescent substrate and quantified using Image Lab software.

### 2.4. Quantitative Real-Time PCR

Total RNA was extracted from the livers and hepatocytes using TRIzol™ Reagent (#15596018; ThermoFisher Scientific) and reversely transcribed to cDNA by the RT First Strand cDNA Synthesis Kit (#G3330; Servicebio, Wuhan, China) according to the manufacturer's instructions. Next, quantitative real-time PCR was performed with SYBR Green Master Mix and normalized to the internal control using the 2^-∆∆Ct^ method [36–39]. The thermocycling conditions were as follows: 95°C for 10, then 40 cycles of 95°C for 2 sec, 60°C for 20 sec, and 70°C for 10 sec. The primer sequences were listed as follows: collagen 1*α*1 (Col1*α*1), forward, 5′-AGGCTTCAGTGGTTTGGATG-3′ and reverse, 5′-CACCAACAGCACCATCGTTA-3′; Col3*α*1, forward, 5′-CCCAACCCAGAGATCCCATT-3′ and reverse, 5′-GAAGCACAGGAGCAGGTGTAGA-3′; connective tissue growth factor (CTGF), forward, 5′-TGTGTGATGAGCCCAAGGAC-3′ and reverse, 5′-AGTTGGCTCGCATCATAGTTG-3′; transforming growth factor-beta 1 (TGF-*β*1), forward, 5′-TGCGCTTGCAGAGATTAAAA-3′ and reverse, 5′-CGTCAAAAGACAGCCACTCA-3′; miR-137-3p, forward 5′-TTATTGCTTAAGAATACGCG-3′ and reverse, 5′-TCGTATCCAGTGCAGGGTC-3′; GAPDH, forward, 5′-CGTGCCGCCTGGAGAAACC-3′ and reverse, 5′-TGGAAGAGTGGGAGTTGCTGTTG-3′; and U6, forward, 5′-CTCGCTTCGGCAGCACA-3′ and reverse, 5′-AACGCTTCACGAATTTGCGT-3′.

### 2.5. Primary Hepatocyte Isolation and Culture

Primary hepatocytes were isolated from 8 week-year-old C57BL/6 mice using a two-step collagenase perfusion method as previously described [27]. Briefly, mice were sacrificed with the livers harvested and perfused with collagenase buffer. Next, the livers were gently shacked to release the hepatocytes into the medium. Then, the cells were washed and purified with a 50% Percoll solution (#17-0891-01; GE Healthcare Life Sciences, Pittsburgh, PA, USA). To mimic NAFLD stimulation in vitro, primary hepatocytes were stimulated with 0.5 mmol/L palmitic acid plus 1.0 mmol/L oleic acid (PO) for 24 h [27]. To verify the role of miR-137-3p, primary hepatocytes were preincubated with miR-137-3p antagomir (50 nmol/L) for 24 h using the Lipofectamine 6000™ reagent (#C0526; Beyotime) before PO stimulation. For PDE4D silence, cells were pretransfected with the small interfering RNA against PDE4D (siPDE4D) or siRNA for 24 h before miR-137-3p antagomir treatment [40–42]. The siPDE4D (#sc-152130) and matched siRNA were purchased from Santa Cruz Biotechnology (Danvers, MA, USA).

### 2.6. Luciferase Reporter Assay

The wild-type (WT) or truncated (TRU) 3′-UTR of PDE4D were constructed and cloned into the pGL3-basic luciferase reporter plasmid (Promega, Madison, Wisconsin, USA), which were then cotransfected with miR-137-3p agomir (50 nmol/L) into HEK293T cells for 48 h. Subsequently, the cells were collected, and the firefly and Renilla luciferase activities were determined using the Dual-Luciferase Reporter Assay System (Promega), with the results expressed as the ratio of firefly to Renilla luciferase activity [43, 44].

### 2.7. Statistical Analysis

Data were expressed as the means ± standard deviations and analyzed using SPSS software (Version 19.0). Comparisons between two groups with a normal distribution and homogeneity of variance were determined by an unpaired two-tailed Student's *t*-test. For multiple comparisons, one-way analysis of variance followed by the Tukey post hoc test was performed. A *p* value < 0.05 was considered significant.

## 3. Results

### 3.1. miR-137-3p Agomir Improves HFD-Induced NAFLD in Mice

**Figure 1:**
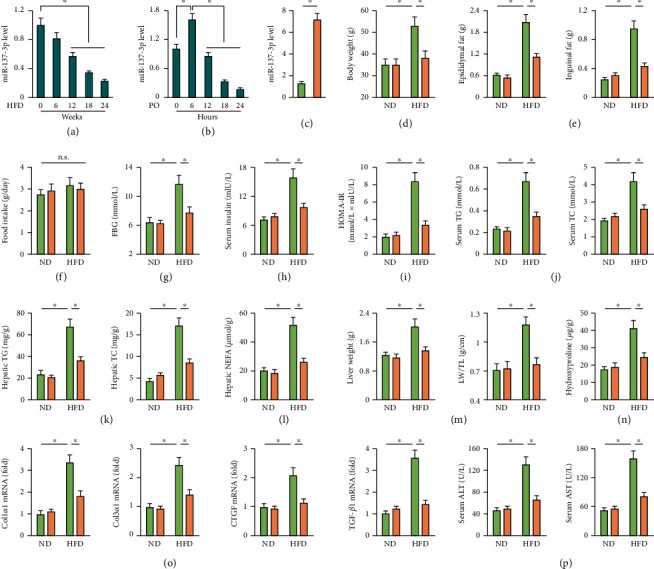
miR-137-3p agomir improves HFD-induced NAFLD in mice. (a) Mice were fed with a HFD for 24 weeks to establish NAFLD, whereas the matched mice were maintained on a ND. Relative miR-137-3p level in the liver from mice was detected (*n* = 6). (b) Primary hepatocytes were isolated from 8 week-year-old C57BL/6 mice and stimulated with 0.5 mmol/L palmitic acid plus 1.0 mmol/L oleic acid (PO) for 24 h. Relative miR-137-3p level in PO-stimulated primary hepatocytes was detected (*n* = 6). (c) For miR-137-3p overexpression, mice were intraperitoneally injected with the miR-137-3p agomir or agomir control (100 mg/kg weekly) for 6 consecutive weeks before being sacrificed. Relative miR-137-3p level in the liver from mice with or without miR-137-3p agomir injection (*n* = 6). (d) Body weight in mice after 24 weeks of HFD feeding (*n* = 8). (e) The weights of epididymal and inguinal fat pad in mice after 24 weeks of HFD feeding (*n* = 8). (f) Food intake (*n* = 10). (g, h) Serum parameters of FBG and insulin in mice (*n* = 6). (i) Quantification of the insulin resistance index HOMA-IR (*n* = 6). (j) Serum parameters of TG and TC in mice (*n* = 6). (k, l) Hepatic lipid accumulation as determined by the TG, TC, and NEFA levels (*n* = 6). (m) Quantification of the liver weight (LW) and LW to tibial length ratio (LW/TL) (*n* = 8). (n) Hepatic hydroxyproline level (*n* = 6). (o) Relative mRNA levels of Col1*α*1, Col3*α*1, CTGF, and TGF-*β*1 in the liver (*n* = 6). (p) Serum ALT and AST levels (*n* = 8). Data were expressed as the means ± standard deviations, and ^∗^*p* < 0.05 was considered significant. n.s. indicated no significance.

We first examined hepatic miR-137-3p expression in mice upon HFD stimulation and observed a significant decrease of miR-137-3p in the liver from HFD-treated mice (Figure 1(a)). Intriguingly, miR-137-3p expression was transiently elevated in primary hepatocytes with PO stimulation at 6 h but significantly decreased at 18 h and 24 h (Figure 1(b)). To clarify the function of miR-137-3p in HFD-induced NAFLD, mice were treated with miR-137-3p agomir (Figure 1(c)). As shown in Figure 1(d), miR-137-3p agomir treatment reduced the body weight of HFD mice. The weight of adipose tissue was also decreased by miR-137-3p agomir, as determined by the decreased weights of epididymal and inguinal fat pads (Figure 1(e)). However, miR-137-3p agomir treatment did not affect food intake either under basal conditions or upon HFD stimulation (Figure 1(f)). Systemic metabolic disorder (e.g., hyperglycemia, insulin resistance, and hyperlipemia) is a key feature of NAFLD and extremely facilitates the progression of hepatic injury [1]. Consistently, HFD-treated mice had higher FBG and serum insulin levels, and the HOMA-IR, an index of insulin resistance, was also increased in HFD mice, which were all reduced by miR-137-3p agomir treatment (Figures 1(g)–1(i)). In addition, HFD-induced elevations of serum TG and TC were also decreased in mice treated with miR-137-3p agomir (Figure 1(j)). In line with the systemic alterations, miR-137-3p agomir also reduced lipid accumulation in the liver upon HFD stimulation, as evidenced by the decreased hepatic TG, TC, and NEFA contents (Figures 1(k) and 1(l)). As expected, HFD-induced gain of liver weight was blocked in mice with miR-137-3p agomir treatment (Figure 1(m)). Hepatic fibrosis is an important pathological change during NAFLD progression, and our data showed that miR-137-3p agomir significantly prevented HFD-induced hepatic fibrosis in mice, as confirmed by the decreased hepatic hydroxyproline content and mRNA levels of fibrotic markers, including Col1*α*1, Col3*α*1, CTGF, and TGF-*β*1 (Figures 1(n) and 1(o)). Moreover, serum ALT and AST levels were also decreased by miR-137-3p agomir treatment, indicating an improved liver function (Figure 1(p)). Collectively, these findings indicate that miR-137-3p agomir improves HFD-induced NAFLD in mice.

### 3.2. miR-137-3p Antagomir Aggravates HFD-Induced NAFLD in Mice

**Figure 2:**
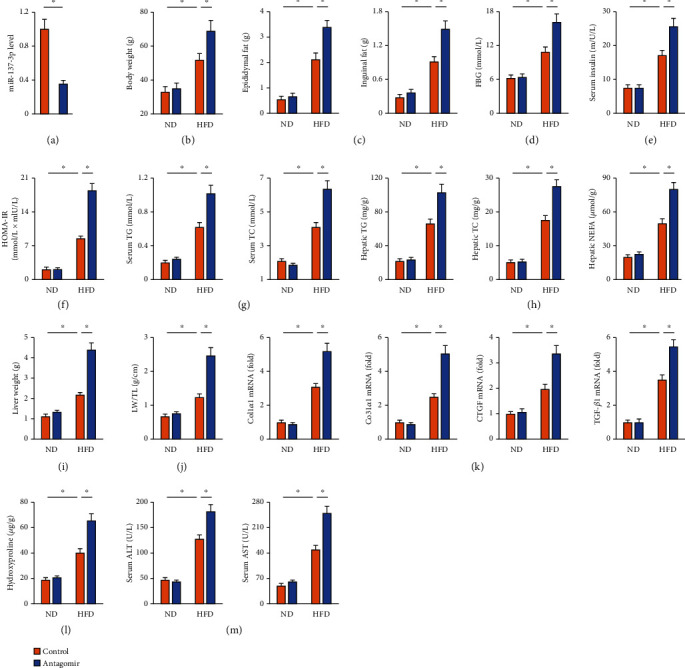
miR-137-3p antagomir aggravates HFD-induced NAFLD in mice. (a) For miR-137-3p inhibition, mice were intraperitoneally injected with the miR-137-3p antagomir or antagomir control (100 mg/kg weekly) for 6 consecutive weeks before being sacrificed. Relative miR-137-3p level in the liver from mice with or without miR-137-3p antagomir injection (*n* = 6). (b) Body weight in mice after 24 weeks of HFD feeding (*n* = 8). (c) The weights of epididymal and inguinal fat pad in mice after 24 weeks of HFD feeding (*n* = 8). (d, e) Serum parameters of FBG and insulin in mice (*n* = 6). (f) Quantification of the insulin resistance index HOMA-IR (*n* = 6). (g) Serum parameters of TG and TC in mice (*n* = 6). (h) Hepatic lipid accumulation as determined by the TG, TC, and NEFA levels (*n* = 6). (i, j) Quantification of the liver weight and LW/TL (*n* = 8). (k) Hepatic hydroxyproline level (*n* = 6). (l) Relative mRNA levels of Col1*α*1, Col3*α*1, CTGF, and TGF-*β*1 in the liver (*n* = 6). (m) Serum ALT and AST levels (*n* = 8). Data were expressed as the means ± standard deviations, and ^∗^*p* < 0.05 was considered significant.

Then, HFD-fed mice were treated with miR-137-3p antagomir and the efficiency is presented in Figure 2(a). As shown in Figures 2(b) and 2(c), miR-137-3p antagomir significantly increased body and adipose tissue weight. HFD-induced systemic hyperglycemia, insulin resistance, and hyperlipemia were also aggravated in the presence of miR-137-3p antagomir (Figures 2(d)–2(g)). In addition, hepatic steatosis was exacerbated in miR-137-3p antagomir-treated mice upon HFD stimulation, as verified by the increased hepatic TG, TC, and NEFA contents (Figure 2(h)). The mice treated with miR-137-3p antagomir also had heavier liver weight (Figures 2(i) and 2(j)). HFD-induced hepatic fibrosis and injury were significantly aggravated in miR-137-3p antagomir-treated mice (Figures 2(k)–2(m)). These data suggest that miR-137-3p antagomir aggravates HFD-induced NAFLD in mice.

### 3.3. miR-137-3p Agomir Reduces Hepatic Oxidative Stress and Inflammation in HFD Mice

**Figure 3:**
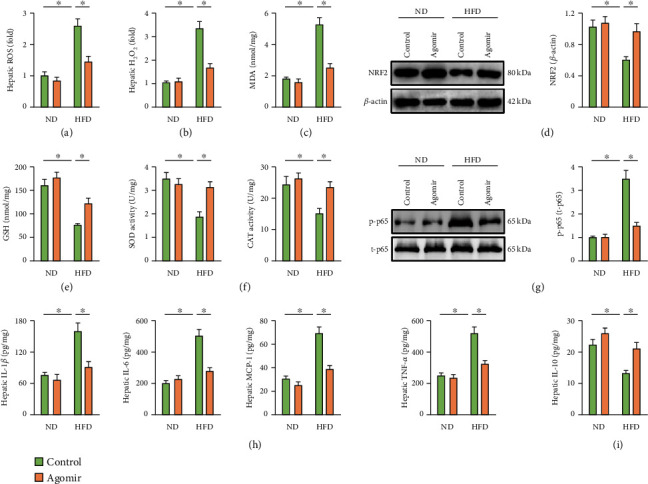
miR-137-3p agomir reduces hepatic oxidative stress and inflammation in HFD mice. (a) Mice were fed with a HFD for 24 weeks to establish NAFLD and were also intraperitoneally injected with the miR-137-3p agomir or agomir control (100 mg/kg weekly) at the last 6 consecutive weeks. Relative hepatic ROS level determined by DCFH-DA probe (*n* = 6). (b) Relative H_2_O_2_ level in the liver (*n* = 6). (c) MDA generation in the liver (*n* = 6). (d) NRF2 protein expression determined by western blot (*n* = 6). (e) GSH level in the liver (*n* = 6). (f) Total SOD and CAT activity in the liver (*n* = 6). (g) p65 phosphorylation determined by western blot (*n* = 6). (h, i) Hepatic IL-1*β*, IL-6, MCP-1, TNF-*α*, and IL-10 levels determined by the commercial ELISA kits (*n* = 6). Data were expressed as the means ± standard deviations, and ^∗^*p* < 0.05 was considered significant.

Oxidative stress is implicated in the pathogenesis of NAFLD [45]. As shown in Figures 3(a) and 3(b), hepatic ROS and H_2_O_2_ levels were significantly elevated in HFD mice, which were reduced by miR-137-3p agomir. Accordingly, HFD-induced lipid peroxidation was suppressed in miR-137-3p agomir-treated mice, as evidenced by the decreased hepatic MDA content (Figure 3(c)). NRF2 is the core regulator of antioxidant defenses through upregulating the downstream antioxidant genes [45, 46]. As shown in Figure 3(d), hepatic NRF2 expression, as well as the antioxidant GSH, total SOD activity, and CAT activity were all decreased upon HFD stimulation, which were significantly prevented in the presence of miR-137-3p agomir (Figures 3(d)–3(f)). Chronic inflammation is the other pathogenic factor of NAFLD [15]. As shown in Figure 3(g), HFD treatment promoted NF-*κ*B activation in the liver that was significantly suppressed by miR-137-3p agomir, as confirmed by the decreased p65 phosphorylation. Consistently, the levels of proinflammatory cytokines, including IL-1*β*, IL-6, MCP-1, and TNF-*α*, were decreased, while the anti-inflammatory IL-10 was increased in the liver from mice with miR-137-3p agomir treatment (Figures 3(h) and 3(i)). Taken together, we conclude that miR-137-3p agomir reduces hepatic oxidative stress and inflammation in HFD mice.

### 3.4. miR-137-3p Antagomir Promotes Hepatic Oxidative Stress and Inflammation in HFD Mice

**Figure 4:**
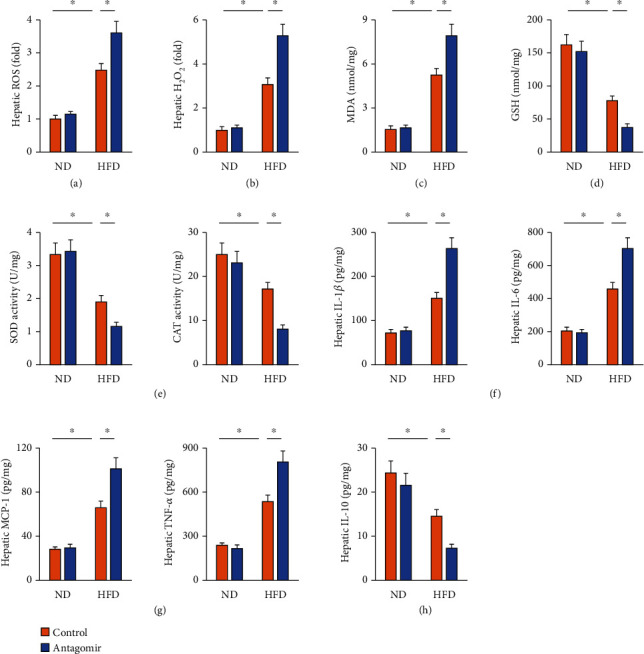
miR-137-3p antagomir promotes hepatic oxidative stress and inflammation in HFD mice. (a) Mice were fed with a HFD for 24 weeks to establish NAFLD and were also intraperitoneally injected with the miR-137-3p antagomir or antagomir control (100 mg/kg weekly) at the last 6 consecutive weeks. Relative hepatic ROS level determined by DCFH-DA probe (*n* = 6). (b) Relative H_2_O_2_ level in the liver (*n* = 6). (c) MDA generation in the liver (*n* = 6). (d) GSH level in the liver (*n* = 6). (e) Total SOD and CAT activity in the liver (*n* = 6). (f–h) Hepatic IL-1*β*, IL-6, MCP-1, TNF-*α*, and IL-10 levels determined by the commercial ELISA kits (*n* = 6). Data were expressed as the means ± standard deviations, and ^∗^*p* < 0.05 was considered significant.

Conversely, miR-137-3p antagomir-treated mice had higher hepatic ROS and H_2_O_2_ generations upon HFD stimulation (Figures 4(a) and 4(b)). As expected, hepatic MDA level was further increased by miR-137-3p antagomir treatment (Figure 4(c)). In contrast, HFD-induced suppressions on hepatic GSH level, total SOD activity, and CAT activity were further amplified by miR-137-3p antagomir (Figures 4(d) and 4(e)). In addition, the extent of hepatic inflammation was more severe in miR-137-3p antagomir-treated mice than those treated with control antagomir in response to HFD stimulation, as evidenced by the increased hepatic IL-1*β*, IL-6, MCP-1, and TNF-*α* levels and decreased IL-10 level (Figures 4(f)–4(h)). Our results identify that miR-137-3p antagomir promotes hepatic oxidative stress and inflammation in HFD mice.

### 3.5. miR-137-3p Agomir Ameliorates HFD-Induced NAFLD through Activating AMPK*α*

**Figure 5:**
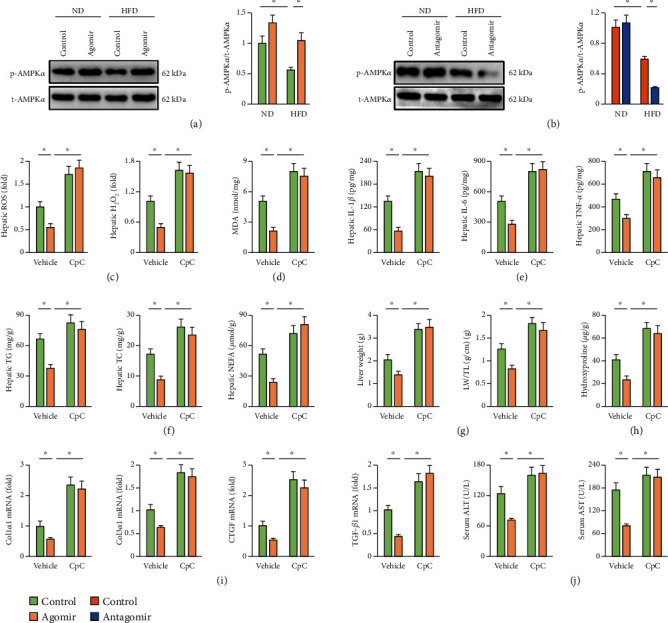
miR-137-3p agomir ameliorates HFD-induced NAFLD through activating AMPK*α*. (a, b) Mice were fed with a HFD for 24 weeks to establish NAFLD and were also intraperitoneally injected with the miR-137-3p agomir, antagomir, or respective controls (100 mg/kg weekly) at the last 6 consecutive weeks. AMPK*α* phosphorylation determined by western blot (*n* = 6). (c) To inhibit AMPK*α*, HFD mice were intraperitoneally injected with 20 mg/kg CpC every other day for 8 consecutive weeks before the mice were sacrificed. Relative hepatic ROS and H_2_O_2_ levels in the liver (*n* = 6). (d) MDA generation in the liver (*n* = 6). (e) Hepatic IL-1*β*, IL-6, and TNF-*α* levels determined by the commercial ELISA kits (*n* = 6). (f) Hepatic lipid accumulation as determined by the TG, TC, and NEFA levels (*n* = 6). (g) Quantification of the liver weight and LW/TL (*n* = 8). (h) Hepatic hydroxyproline level (*n* = 6). (i) Relative mRNA levels of Col1*α*1, Col3*α*1, CTGF, and TGF-*β*1 in the liver (*n* = 6). (j) Serum ALT and AST levels (*n* = 8). Data were expressed as the means ± standard deviations, and ^∗^*p* < 0.05 was considered significant.

Next, we tried to investigate the potential involvement of AMPK*α* in miR-137-3p agomir-mediated hepatoprotective effects against NAFLD. As shown in Figures 5(a) and 5(b), miR-137-3p agomir was preserved, while miR-137-3p antagomir significantly reduced hepatic AMPK*α* phosphorylation upon HFD stimulation. Then, HFD mice were treated with CpC to inhibit AMPK*α* to further validate its necessity in this process. As shown in Figures 5(c) and 5(d), AMPK*α* inhibition significantly abolished the antioxidant effects of miR-137-3p agomir in NAFLD mice, as determined by the increased hepatic ROS, H_2_O_2_, and MDA generation. In addition, miR-137-3p agomir-mediated suppression on hepatic inflammation was also abrogated by CpC (Figure 5(e)). Furthermore, miR-137-3p agomir reduced hepatic lipid accumulation in vehicle-treated HFD mice, yet failed to do so in those with CpC injection (Figure 5(f)). As expected, the loss of liver weight seen in miR-137-3p agomir-treated HFD mice was also prevented by CpC administration (Figure 5(g)). Meanwhile, AMPK*α* inhibition significantly blocked the antifibrotic effects of miR-137-3p agomir in HFD mice, as evidenced by the increased hepatic hydroxyproline content and mRNA levels of fibrotic markers (Figures 5(h) and 5(i)). More importantly, CpC-treated HFD mice showed higher serum ALT and AST levels in the presence of miR-137-3p agomir, indicating that miR-137-3p agomir-mediated hepatoprotective effects against NAFLD were blunted by AMPK*α* inhibition (Figure 5(j)). Together, we prove that miR-137-3p agomir ameliorates HFD-induced NAFLD through activating AMPK*α*.

### 3.6. miR-137-3p Activates AMPK*α* through Downregulating PDE4D

**Figure 6:**
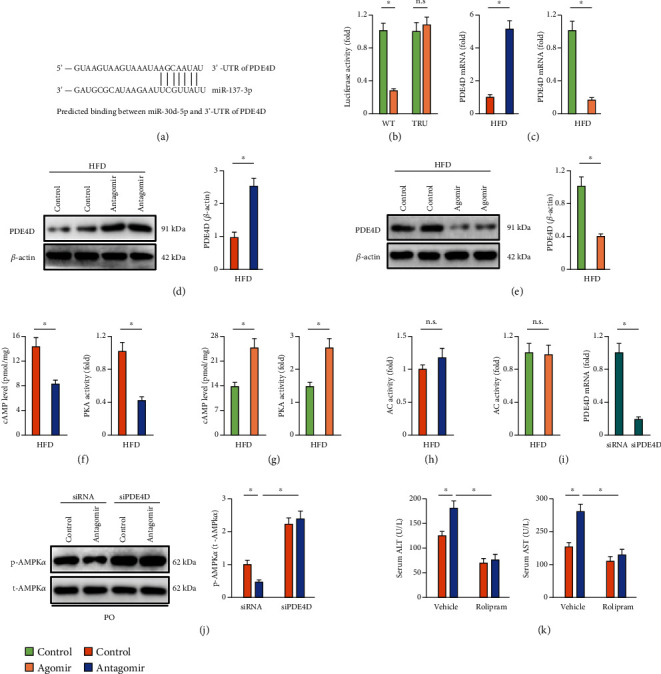
miR-137-3p activates AMPK*α* through downregulating PDE4D. (a) Graphic representation of the miR-137-3p binding motifs within the 3′-UTR of PDE4D. (b) HEK293T cells were cotransfected the WT or TRU 3′-UTR of PDE4D with miR-137-3p agomir (50 nmol/L) for 48 h, and then, the cells were collected and the luciferase activity was determined using the Dual-Luciferase Reporter Assay System (*n* = 8). (c–e) Mice were fed with a HFD for 24 weeks to establish NAFLD and were also intraperitoneally injected with the miR-137-3p agomir, antagomir, or respective controls (100 mg/kg weekly) at the last 6 consecutive weeks. Relative PDE4D mRNA and protein levels in HFD mice treated with miR-137-3p agomir, antagomir, or their respective controls (*n* = 6). (f, g) The cAMP level and PKA activity in HFD mice treated with miR-137-3p agomir, antagomir, or their respective controls (*n* = 6). (h) Relative AC activity (*n* = 6). (i) Primary hepatocytes were transfected with siPDE4D or siRNA for 24 h, and relative PDE4D mRNA level was detected (*n* = 6). (j) Primary hepatocytes were preincubated with miR-137-3p antagomir (50 nmol/L) for 24 h and then stimulated with PO for 24 h. To knock down PDE4D, cells were pretransfected with siPDE4D or siRNA for 24 h before miR-137-3p antagomir treatment. AMPK*α* phosphorylation in miR-137-3p antagomir-treated primary hepatocytes with or without PDE4D silence (*n* = 6). (k) Mice were fed with a HFD for 24 weeks to establish NAFLD and were also intraperitoneally injected with the miR-137-3p agomir, antagomir, or respective controls (100 mg/kg weekly) at the last 6 consecutive weeks. To inhibit PDE4D, mice were intraperitoneally injected with 0.5 mg/kg rolipram or an equal volume of vehicle every other day for 8 consecutive weeks before the mice were sacrificed. Serum ALT and AST levels in miR-137-3p antagomir-treated HFD mice with or without PDE4D inhibition (*n* = 8). Data were expressed as the means ± standard deviations, and ^∗^*p* < 0.05 was considered significant. n.s. indicated no significance.

Finally, we investigated the potential mechanism through which miR-137-3p activated AMPK*α*. Using the online TargetScan software, PDE4D was selected for further examination because of its role in promoting cAMP hydrolysis and subsequently inactivating the cAMP/PKA pathway, the classic upstream mechanism of AMPK*α* [47, 48]. As shown in Figure 6(a), a predicted binding between miR-137-3p and the 3′-UTR of PDE4D was found. And the results from luciferase reporter assay further validated this direct interaction (Figure 6(b)). Moreover, we found that miR-137-3p antagomir increased, while miR-137-3p agomir decreased hepatic PDE4D mRNA and protein levels in HFD mice (Figures 6(c)–6(e)). As expected, hepatic cAMP level and PKA activity were decreased by miR-137-3p antagomir, yet increased by miR-137-3p agomir in HFD mice (Figures 6(f) and 6(g)). AC catalyzes the generation of cAMP from ATP; however, our data suggested that neither the antagomir nor the agomir of miR-137-3p affected hepatic AC activity in HFD mice (Figure 6(h)). To confirm the necessity of PDE4D in miR-137-3p antagomir-mediated inactivation of AMPK*α*, primary hepatocytes were pretransfected with siPDE4D to knock down the endogenous PDE4D expression (Figure 6(i)). As shown in Figure 6(j), miR-137-3p antagomir significantly reduced AMPK*α* phosphorylation in PO-treated hepatocytes, which was prevented in those with PDE4D silence. In addition, we also used a specific PDE4D inhibitor, rolipram, to suppress PDE4D activity in HFD mice. As expected, miR-137-3p antagomir-mediated deleterious effects on NAFLD were attenuated by rolipram, as evidenced by the decreased serum ALT and AST levels (Figure 6(k)). In conclusion, we reveal that miR-137-3p activates AMPK*α* through downregulating PDE4D.

## 4. Discussion

The liver is one of the most important organs to modulate glycolipid metabolism and the whole-body energy homeostasis, whose function would be extremely impaired due to the excessive hepatic lipid accumulation. NAFLD consists of a spectrum of pathophysiological conditions that ranges from simple hepatic steatosis to advanced NASH associated with enhanced hepatic inflammation and fibrogenesis, then to the end-stage liver disease such as cirrhosis and hepatocellular carcinoma [1, 49]. Due to the high prevalence of metabolic syndrome nowadays, NAFLD has become one of the most common chronic liver diseases and affects over 25% of the global population worldwide [50]. Currently, no specific and effective therapies are available to prevent NAFLD progression, and transplantation is the only choice for these patients with end-stage NAFLD. In the present study, we found that hepatic miR-137-3p expression was significantly decreased in the development of NAFLD and that the treatment with miR-137-3p agomir could reduce hepatic oxidative stress and inflammation, thereby improving NAFLD in mice. Taken together, our findings identify miR-137-3p as a promising therapeutic target to treat NAFLD.

The exact mechanisms for the initiation and progression of NAFLD remain unclear. Emerging studies report that excessive hepatic ROS and chronic inflammation are closely associated with hepatocyte injury and hepatic fibrosis. Under physiological conditions, the liver contains multiple antioxidant defenses, including the antioxidant enzymes (e.g., SOD and CAT), which catalyze the transition from nocuous free radicals to innocuous water. Yet, the expressions of these enzymes in the liver were significantly reduced in the context of NAFLD [46, 51]. NRF2 is a redox-sensitive transcription factor to induce the expression of these antioxidant enzymes, and our data revealed that hepatic NRF2 expression was significantly downregulated in HFD mice, accompanied with decreased GSH content, and total SOD and CAT activities. NF-*κ*B functions as the major modulator of inflammatory response and is sequestered in the cytoplasm as an inactive form through binding to the inhibitor of NF-*κ*B in quiescent cells, which then translocates to the nucleus to promote the transcription of various inflammatory cytokines under stressed conditions [52]. Herein, we found that NF-*κ*B p65 phosphorylation was enhanced in the liver from HFD mice, and the levels of downstream IL-1*β*, IL-6, MCP-1, and TNF-*α* were also increased. Intriguingly, miR-137-3p agomir significantly reduced, whereas miR-137-3p antagomir further promoted hepatic oxidative stress and inflammation upon HFD stimulation.

miRNAs are a class of endogenous genetic regulators with pleiotropic functions, such as controlling ROS production and inflammatory response. Cheng et al. demonstrated that miR-421 overexpression significantly decreased hepatic MnSOD and CAT expression and subsequently aggravated oxidative damage upon NAFLD stimulation via directly targeting sirtuin 3 [53]. In addition, Zhang et al. recently showed that miR-96-5p was capable of suppressing p66shc expression, thereby reducing oxidative stress and hepatic steatosis [54]. Based on these contexts, we thus investigated the function of miR-137-3p, a potential antioxidant molecule in the liver, during NAFLD progression [26]. Intriguingly, our data revealed that miR-137-3p overexpression could reduce hepatic oxidative stress and inflammation, thereby improving NAFLD in mice. Mechanistically, miR-137-3p is directly bound to the 3′-UTR of PDE4D, a cAMP-specific phosphodiesterase, and subsequently increased hepatic cAMP level and PKA activity, thereby activating the downstream AMPK*α* pathway. Consistently, other findings also proved that the treatment with PDE inhibitors could protect against HFD-induced NAFLD [55, 56]. Beyond AMPK*α*, many other downstream targets of miR-137-3p have been proposed. Tian et al. revealed that miR-137-3p directly bound to the 3′-UTR of Src and subsequently inhibited the MAPK pathway [23]. Findings from Wei et al. identified AFM as a potential target of miR-137-3p to mediate its regulation on the progression of hepatocellular carcinoma [24]. More recently, Yang et al. reported that miR-137-3p negatively regulated peptidylprolyl isomerase C (Ppic) expression via direct targeting and that Ppic knockdown partially reversed the effects of miR-137-3p inhibition [57]. Whether other possible targets of miR-137-3p exist in the regulation of NAFLD remains unclear and needs further investigation.

In summary, we prove that miR-137-3p is a key player in regulating NAFLD via activating AMPK*α*.

## Data Availability

The data that support the findings of this study are available from the corresponding author upon reasonable request.
